# Identification of Microbial Profiles in Heavy-Metal-Contaminated Soil from Full-Length 16S rRNA Reads Sequenced by a PacBio System

**DOI:** 10.3390/microorganisms7090357

**Published:** 2019-09-16

**Authors:** Moonsuk Hur, Soo-Je Park

**Affiliations:** 1 Microorganism Resources Division, National Institute of Biological Resources, 42 Hwangyeong-ro, Incheon 22689, Korea; heomsk@korea.kr; 2 Department of Biology, Jeju National University, 102 Jejudaehak-ro, Jeju 63243, Korea

**Keywords:** heavy metals, soil, PacBio, 16S rRNA gene, mines

## Abstract

Heavy metal pollution is a serious environmental problem as it adversely affects crop production and human activity. In addition, the microbial community structure and composition are altered in heavy-metal-contaminated soils. In this study, using full-length 16S rRNA gene sequences obtained by a PacBio RS II system, we determined the microbial diversity and community structure in heavy-metal-contaminated soil. Furthermore, we investigated the microbial distribution, inferred their putative functional traits, and analyzed the environmental effects on the microbial compositions. The soil samples selected in this study were heavily and continuously contaminated with various heavy metals due to closed mines. We found that certain microorganisms (e.g., sulfur or iron oxidizers) play an important role in the biogeochemical cycle. Using phylogenetic investigation of communities by reconstruction of unobserved states (PICRUSt) analysis, we predicted Kyoto Encyclopedia of Genes and Genomes (KEGG) functional categories from abundances of microbial communities and revealed a high proportion belonging to transport, energy metabolism, and xenobiotic degradation in the studied sites. In addition, through full-length analysis, *Conexibacter*-like sequences, commonly identified by environmental metagenomics among the rare biosphere, were detected. In addition to microbial composition, we confirmed that environmental factors, including heavy metals, affect the microbial communities. Unexpectedly, among these environmental parameters, electrical conductivity (EC) might have more importance than other factors in a community description analysis.

## 1. Introduction

As of 2017, 4677 out of 5544 mines (for coal, metal, and nonmetals) in Korea have been abandoned owing to environmental concerns and/or economic factors. In particular, in the case of metal mines, most mines (about 95%; 2084 out of 2184) in Korea are in disuse due to soil pollution caused by waste rock and mine tailings with acid mine drainage (AMD) [1]. The mine tailings dams and/or AMD contaminated by heavy metals, including iron and cadmium, have led to serious environmental problems related to crops and public health through the contamination of the water supply and food chain, respectively. Although these contaminated areas have been reclaimed by a long-term plan under the Ministry of Environment in Korea, there are rising concerns regarding soil contamination by heavy metals [2,3].

Heavy metal pollution by anthropogenic activities affects microbial activities and community structures in terrestrial environments. There is a specific relationship between microorganisms and minerals in these extremely toxic environments. Soil microorganisms can affect plant growth and increase the accumulation of heavy metals in plants (i.e., phytoremediation) [4,5]. Therefore, the analysis of microbial communities might provide fundamental information for phytoextraction improvement [6]. Most previous studies have focused on identifying microbial community structures in polluted soils or isolating useful microorganisms for the removal of heavy metals see review in [7]. Moreover, based on cultivation and metagenome approaches, we can easily understand and expand our knowledge of new microbe‒mineral interactions [8,9,10]. Recently, microbial communities from various environments have been extensively sequenced and analyzed by next-generation sequencing (NGS) platforms using partial regions of the 16S rRNA gene (e.g., V1‒V3, V3‒V4, V4‒V5, or V4‒V6) [5,11,12,13,14,15,16,17]. Additionally, numerous unrecognized bacteria and archaea have been identified by NGS technology, and the results can serve as fundamental taxonomic information in (meta)genome analysis with putatively deduced functional characterizations [18,19].

Although previous studies using specific regions of the 16S rRNA gene have shown that taxonomic assignments are highly sensitive, advanced high-throughput analysis revealed unintended missing classifications with less accuracy, especially at the genus or species level [20]. This might be because most of the naturally existing microorganisms have not yet been cultivated and identified.

Recently, a single molecular real-time (SMRT) DNA sequencing system has been developed by Pacific Biosciences (PacBio) and applied to microbial community and (meta)genome analyses [21,22,23,24]. This system is able to generate raw reads more than 10 kb long with a low error rate [25]. Although the PacBio platform is less attractive than other short-read platform such as Illumina and Ion-Torrent due to the higher cost, it has been applied to microbial community analysis without primer bias and with high quality [26,27,28]. Based on the advantages of the PacBio platform, we also expect to obtain more accurate results from full-length reads generated by the PacBio system for the microbial community in heavy-metal-contaminated soil (from disused mines) and identify the rare microorganism(s) in the biosphere [29]. As mentioned above, most mines in Korea are in disuse and crops and human health might be affected by contaminated water. Additionally, very little is known about the composition and structure of the bacterial community in these contaminated areas in Korea. Therefore, in the present study, we characterized and compared the microbial communities in heavy-metal-contaminated sites in the Korea. The main objectives of the present study are to demonstrate the practical application of the PacBio system in microbial community structure analysis and to investigate the microbial diversity and structure, along with the effect of environmental parameters, including heavy metals, on them.

## 2. Materials and Methods

### 2.1. Sample Collection and Characterization

For the microbial community analysis, we selected three sites in regions with heavy metal contamination, namely, Hwaseong (H), Daegu (D), and Bonghwa (B), in the Korea (Table S1). From each site, duplicate soil samples were collected from the surface (2–5 cm depth; F) and subsurface (60–75 cm depth; B) at five randomly selected spots (1–5) located 1 m from each other for replication [30]. Alphanumeric codes were assigned to each sample; for example, BF1 is the first surface sample collected at Bonghwa. The samples were transferred to sterile plastic tubes or bags and stored at ‒80 °C until the microbial community structure analysis. The sampling depths were determined based on the recovery depth for heavy-metal-contaminated soil from abandoned mines in the Korea [31]. Inductively coupled plasma atomic emission spectroscopy (ICP-OES; PerkinElmer Optima 7300 DV) was used to determine the concentrations of the heavy metals [copper (Cu), lead (Pb), arsenic (As), zinc (Zn), and cadmium (Cd)]. Before the analysis, the soil samples were sequentially filtered through 2.0-mm and 0.15-mm sieves, dried, and then digested with concentrated nitric acid. Calibration was performed using a distilled water blank and standard solution [31,32]. The concentrations of the selected heavy metals in each sample were determined in duplicate. Total nitrogen (TN), total carbon (TC), and organic matter (OM) were measured using a CNS analyzer (US/Vario Max CN, Elementar Analysensysteme, GmbH, Hanau, Germany). pH and electrical conductivity (EC) were determined using a pH/EC meter (Sevenmulti S40, Mettler Toledo, Greifensee, Switzerland) [33]. In situ temperature was determined using a thermometer (waterproof digital thermocouple thermometer, A1.T9234, Daihan, Seoul, Korea). The particle size distribution of the soil samples was plotted on a particle size distribution curve (semi-log graph; Table S1) to assess soil texture [33,34] (Table S1).

### 2.2. PCR Amplification of Bacterial 16S rRNA Gene and Sequencing by PacBio System

Total genomic DNA (gDNA) was extracted from each prepared sample using a Power Soil DNA kit (Mo Bio Laboratories, Solanan Beach, CA, USA). The quality and quantity of the extracted gDNA were determined using a DS-11 Plus Spectrophotometer (DeNovix, Inc., Wilmington, DE, USA) and by performing electrophoresis on a 1.0% (w/v) agarose gel, respectively.

For full-length bacterial 16S rRNA gene amplification, we performed PCR using the following mixture: 10 μL of 2× Dr. MAX Master Mix Solution (Doctor Protein Corp., Seoul, Korea), 1 μM of 27F (5’-AGRGTTYGATYMTGGCTCAG-3’) and 1492R (5’-RGYTACCTTGTTACGACTT-3’) primer set (final concentration), and ~10 ng of environmental DNA as a template. Where necessary, the template was diluted using 0.1× TRIS-EDTA buffer to decrease the concentration of PCR inhibitors. Cycling was performed with an initial denaturation at 95°C for 7 min, followed by 30 cycles at 95 °C for 30 s, 55 °C for 30 s, and 72 °C for 30 s, and a final extension at 72 °C for 10 min. The detailed sequences for barcode and adapter are provided in Table S2. Amplification products were separated by agarose gel electrophoresis and purified using a PCR Clean-up Kit (LaboPass, Cosmo Genetech, Seoul, Korea). SMRTbell adapters were then ligated onto the purified PCR products and the libraries were sequenced by Pacific Biosciences using P6-C4 chemistry on a PacBio RS II SMRT DNA sequencing system (Pacific Biosciences, Menlo Park, CA, USA).

### 2.3. Sequence Analysis, Diversity Indices, and Statistics

Raw sequences were initially processed through the PacBio SMRT portal. All purified sequencing data were then processed using Mothur version 1.39.5 [24,35,36]. All sequences were aligned against a SILVA-based reference alignment and classified against the greengenes (version gg_13_8_99) reference database using a negative Bayesian classifier implemented within Mothur [36]. Diversity indices (Shannon and Simpson indices, and the Chao1 nonparametric richness index (and Unweighted Pair Group Method with Arithmetic Mean (UPGMA) clustering, nonmetric multidimensional scaling (NMDS), and analysis of similarities (ANOSIM) were determined using the Mothur package [37]. A dissimilarity level of 3% was used in further investigations including diversity indices. Habitat specialization was calculated as described by Liu et al. [38] using Levin’s niche breadth index (*B*) [39], which assigns low niche breadth values to specialists (< 1.5) and higher values to generalists (> 3).

A Mann–Whitney *U* test was performed to compare the diversity indices between the microbial communities from each analyzed site. To estimate the relationship between microbial community and environmental factors, a canonical correspondence analysis (CCA) was performed using the VEGAN package [40,41,42]. The 32 highest-ranked genera (>5% proportion in each sample) were assessed using CCA. Manual selection of environmental parameters through application of a Monte Carlo permutation test (999 random permutations) was conducted to determine their statistical significance (*p* < 0.05). The resulting ordination biplot approximated the weight of each OTU with respect to each environmental variable, represented as arrows. The length of the arrows indicated the relative importance of environmental factors that explained variations in the microbial communities. All figures were generated by R packages [43] and/or Origin Pro 2018 (OriginLab, Northampton, MA, USA) [44]. Further, for estimation of the interactions between microbial compositions and environmental parameters, Spearman’s rank correlation coefficient (ρ) was calculated simultaneously using the ‘rcorr’ function with the Hmics package [45] in R to analyze the significance of the correlation. A high correlation coefficient (|ρ| ≥ 0.7) with a *p*-value < 0.05 between microbial compositions and environmental parameters was visualized via Cytoscape (v.3.6.1) [46]. To estimate correlation values between environmental parameters, we used Spearman’s rank correlation analysis [47]. Functional profiles of microbial communities were predicted by phylogenetic investigation of communities by reconstruction of unobserved states (PICRUSt) [13]. Although functional predictions were assigned up to all Kyoto Encyclopedia of Genes and Genomes (KEGG) orthology (KO) numbers, only xenobiotic biodegradation and metabolism, energy metabolism, and membrane transport were selected as contamination-related categories for analysis simplification and clarity. The categories related to “human disease” or “eukaryotes” were excluded due to the lack of relevance to environmental samples.

The full 16S rRNA gene sequences recovered in this study have been deposited in the DDBJ/ENA/GenBank Sequence Read Archive (SRA) under the accession number SRP137440, and are provided as a supplementary file.

## 3. Results

### 3.1. Environmental Data

The geographical and physiochemical parameters of 30 soil samples from the three sites are summarized in Table S1. A pH range of 2.8–6.9 (average pH 4.9 ± 1.3) indicated acidic to near-neutral pH. The subsurface (pH 4.3 ± 1.5) was slightly more acidic than the surface (pH 5.4 ± 1.2). The temperature reached an average of 23 ± 3.0 °C. The EC was in the range of 0.15–20.2 (average: 4.3 ± 5.5) ds/m. Except for the Bonghwa samples (11.1 ds/m), most samples had extremely low EC values (0.86 ds/m). In addition, we found significant differences in the other environmental factors (e.g., TN, OM, and temperature) between sampling site and depth. The average concentrations (ppm) of the major heavy metals (Cu, Pb, As, Zn, and Cd) were estimated as 322.6 ± 320.4, 1175.6 ± 1770.9, 5373.7 ± 9638.3, 3884.6 ± 7491.6, and 28.4 ± 51.7 ppm, respectively (Table S1). In particular, the concentration of As was higher than that of other heavy metals. However, in Hwaseong, the heavy metal concentrations were extremely low and As was undetectable. Although the soil texture was similar, the difference in the soil composition (e.g., sand, silt, and clay) was significant between samples from different sites (Table S1).

### 3.2. General Statistics for 16S rRNA Gene Amplification

A total of 122,702 sequences were obtained and analyzed from the 148,594 raw sequences after quality filtering supplied in the Mothur program (Table 1). The data analyzed for each sample ranged in size from 1490 sequences for sample HF4 to 8284 for sample DB5.

The whole analyzed sequences were classified into different well-determined, candidate, and unclassified phyla (Figure S1a). From all samples, the phylum Proteobacteria was identified as the highly accounted-for (i.e., dominant) group (comprising about 41% of the total number of purified sequences), followed by Acidobacteria, Actinobacteria, Chloroflexi, AD3, Firmicutes, Planctomycetes, Nitrospirae, and Gemmatimonadetes, comprising 19.7%, 10.2%, 7.5%, 5.4%, 4.8%, 3.6%, 2.8%, and 1.3% of all sequences, respectively.

**Figure 1:**
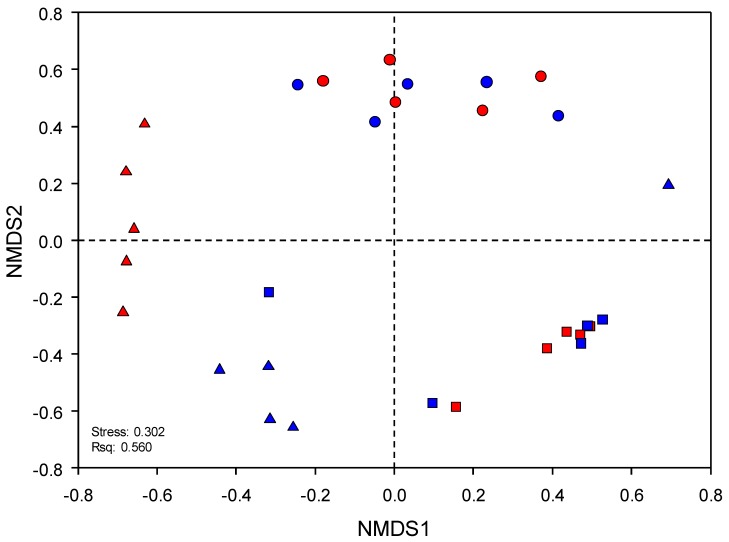
Microbial community profiles of the samples from Hwaseong (circles), Daegu (squares), and Bonghwa (triangles) based on non-metric multidimensional scaling (NMDS) using the Mothur package. Distance matrix was calculated using the Yue and Clayton theta supplied in the Mothur package. Operational taxonomic units (OTUs) were determined based on 3% dissimilarity of nucleotide sequences. Blue and red denote surface and subsurface, respectively.

The results of NMDS showed that microbial communities were apparently clustered by sampling site (Rsq: 0.560 and Stress: 0.302), excluding two samples (DF1 and BF1) (Figure 1). The stress value for NMDS indicated poor representation for microbial communities; however, the ANOSIM results indicated that the microbial community compositions (based on the 97% similarity level) were significantly different between most analyzed samples (Table S3). Furthermore, with the exception of the DF1 sample, UPGMA showed that samples from the three distantly located sites formed a separate cluster (Figure S2). In the case of Bonghwa, we found a tendency to split into two parts depending on the sampling depth. In addition, the samples from Hwaseong formed a separate and distant cluster outside the other samples, indicating a more distinguished relationship.

**Figure 2:**
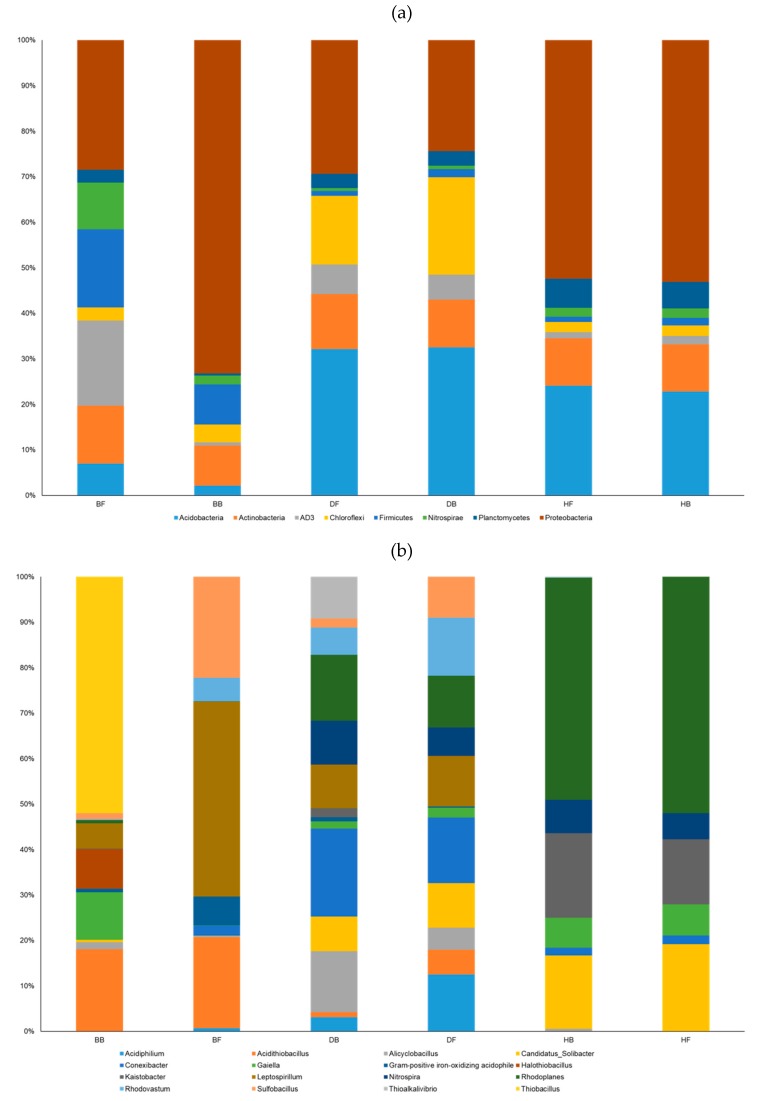
Relative abundance of the most abundant eightbacterial phyla (**a**) and 16 genera (**b**) in the contaminated soil samples. At the genus level, only those with proportions above 5% of the total reads in each sample are shown.

The analyzed sequences were affiliated with 42 phyla and the eight most abundant phyla (>5% proportion of all reads in each sample) were designated as major phyla: Acidobacteria, Actinobacteria, AD3, Chloroflexi, Firmicutes, Nitrospirae, Planctomycetes, and Proteobacteria (Figure 2a and Figure S1). These phyla contributed from 87.4% to 99.7% (average 95.0%) of all sequences. In particular, among the phyla, we found that Proteobacteria accounted for the highest proportion, especially in the BB sample, which had the greatest proportion (about 70%). On the other hand, the proportion of Acidobacteria of the BB sample was less than that of the other sites.

At the genus taxonomic level, the aforementioned sequences were assigned to 720 genera, including the unclassified group with a high taxonomic level (e.g., class, order, or family). For further analysis, we analyzed only 352 clearly assigned genera, excluding the sequences assigned as “unclassified,” although we might have lost many sequences (Figure 2b). Finally, we selected 32 genera from each sample based on >5% of total sequences for the following analyses such as CCA and interaction. Moreover, from a combined sample (as grouped together by the same depth of each site), we found only 16 genera (>5% proportion of each combined sample) designated as dominant microbes (Figure S1b). In this analysis, eight genera, *Leptospirillum*, *Rhodoplanes*, *Thiobacillus*, *Acidithiobacillus*, *Sulfobacillus*, *Conexibacter*, *Candidatus* Solibacter, and *Rhodovastum*, had the highest relative abundance, accounting for about 80% of total bacterial abundance from all samples. The genera *Acidothiobacillus* and *Sulfobacillus* were only identified in two samples (BF and BB) and in BF samples, respectively, as major taxa (Figure 2b). Iron-oxidizing gram-positive acidophiles were identified in Daegu and Hwaseong samples as a minor taxon (less than 0.5% of total bacterial abundance); however, this iron-oxidizer was detected in BF with comparatively high abundance (5% of total bacterial abundance). Unexpectedly, the genus *Halothiobacillus,* isolated from marine environments including hydrothermal vents and considered as an obligately chemolithoautotrophic and sulfur oxidizer, was identified in BB.

Subsequently, to determine generalist and specialist genera among those selected in this study (*n* = 32), we calculated niche breadth (*B*) by Levin’s index [38,39] from all analyzed sites and depths. From this analysis, only 16 genera were determined as generalists (*B* > 3, *Alicyclobacillus*, *Aquicella*, *Clostridium*, *Conexibacter*, *Nitrospira*, and *Rhodanobacter*) and specialists: (*B* < 1.5, *Gallionella*, gram-positive iron-oxidizing_acidophile, *Halothiobacillus*, *Leptolyngbya*, *Leptospirillum*, *Rhodoferax*, *Sulfobacillus*, *Thioalkalivibrio*, *Thiobacillus*, and YNPFFP6 classified into the family Sulfobacillaceae). 

In Table 1, the metrics for alpha diversity (number of OTUs, Chao1 richness, Shannon evenness, Simpson diversity, and Goods’ coverage) of the microbial community are summarized. OTUs were defined as sequences with 97% sequence similarity. While variations in diversity indices were observed, there were no significant differences between surface and subsurface for intragroup and intergroup samples (estimated by Mann‒Whitney *U* test). However, diversity indices of the H (Hwaseong) samples combined from surface and subsurface showed higher values than those of other samples (Figure S4). In particular, the Simpson diversity index showed extremely remarkable differences (*p* = 0.0003 estimated by Mann‒Whitney *U* test). On the other hand, the diversity indices of the samples from Bonghwa and Daegu showed similar ranges (*p* > 0.11 estimated by Mann‒Whitney *U* test) (Figure S4).

### 3.3. Environmental Factors Significantly Affecting the Microbial Community

**Figure 3:**
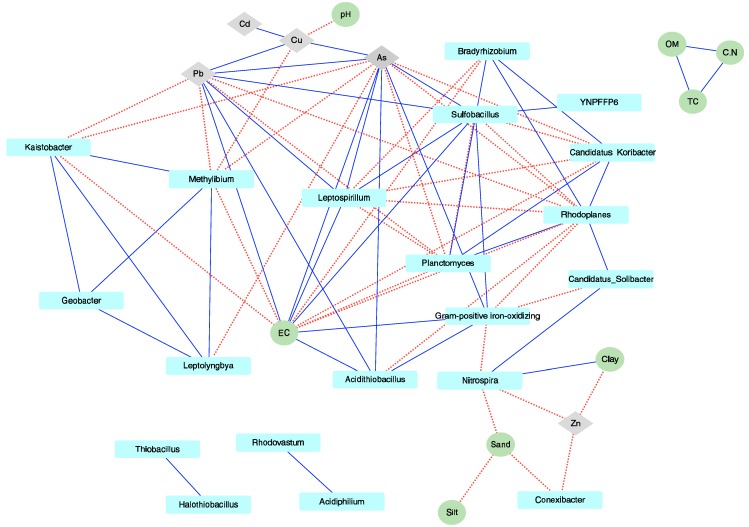
Profile clustering Cytoscape network visualization of the 32 most abundant genera with environmental correlation (solid line, positive correlation; dotted line, negative correlation) estimated by Spearman’s rank analysis. Analyzed genus, heavy metals, and other environmental factors are marked with cyan squares, gray diamonds, and green circles, respectively.

To explain the variation in the microbial communities (i.e., the selected 32 genera) between sampling sites, CCA and association network analysis were performed. These analyses facilitated the investigation of the effect of environmental parameters including soil components. The CCA results indicated that with the exception of the pH and clay, most environmental variables exhibited significant effects on the microbial communities (*p* < 0.012 based on the 999 permutations) (Figure S3). On the *x* axis (CA1), OM, TC, EC, and C/N showed highly positive positions (0.93–0.97), while temperature, pH, and silt showed highly positive positions (0.90–0.99) on the *y* axis (CA2). To estimate the interactions between the microorganism(s) and environmental parameters, an associated network analysis was performed based on Spearman’s rank correlation coefficient (ρ) (Figure 3). From the network analysis, 20 genera and 13 environmental factors were obtained based on the criteria described in the Materials and Methods. Furthermore, a total of 76 correlations (41 positive and 35 negative) were established between environmental factors and genera (Figure 3). Based on this analysis, we found that As and EC, among the environmental factors, and *Rhodoplanes* genus, in the microbial composition, have higher interactions than others. Unexpectedly, four genera (*Leptospirillum*, *Sulfobacillus*, *Acidithiobacillus*, and Gram-positive iron-oxidizer) have positive interactions with EC, but not pH, and it was observed that pH exhibited a negative relationship only with Cu. These genera have been categorized as an acidophilic bacterial group [48]. In addition, four genera (*Thiobacillus*, *Halothiobacillus*, *Rhodovastum*, and *Acidiphilum*) and three environmental variables (TC, C/N, and OM) showed only an intragroup relationship (Figure 3).

### 3.4. Predictive Functional Profiles from Microbial Communities

Although we tried to understand microbial functional traits by analyzing the abundances and distribution of microorganisms in each site, it is difficult to predict their ecological roles. Therefore, to observe and compare study sites, putative functional profiles from 16S rRNA gene sequences were predicted via KEGG pathways of PICRUSt analysis. Among all KEGG pathways predicted by PICRUSt, the abundance of the KEGG category related to “Transport” was estimated as the highest (11.8–13.3% of total KEGG categories), after exclusion of the poorly relevant categories (see Materials and Methods). The proportions for xenobiotic biodegradation and energy metabolism were estimated to be 6.0% and 7.5%, respectively. Notably, between the studied sites, variation in some functional traits of third-tier KO was observed, such as in degradation of DDT, aminobenzoate, and nitrotoluene (Figure S5). However, the proportions of other third-tier functional categories were similar between sampling sites.

## 4. Discussion

Over the past 10 years, NGS technology has been introduced and developed, and has played a central role in the field of microbial ecology for sequencing small-subunit ribosomal RNA genes (e.g., the 16S rRNA gene). Traditional strategies, i.e., clone-based sequencing and culture-dependent methods, for microbial community analysis have recently been extensively replaced by NGS platforms such as pyrosequencing (of Roche 454), paired-end sequencing (HiSeq or MiSeq of Illumina), and an ion semiconductor (IonTorrent). These platforms are now commonly used to generate hundreds of thousands of read sequences from various environmental samples from an amplicon of the variable region(s) of the 16S rRNA gene [49,50]. However, it is well known that the amplicon approach might have an amplification bias that occurs by variable region selection associated with the primer choice [51]. The bias can affect the results for taxonomic classification and diversity indices [52]. Contrastingly, full-length 16S rRNA gene sequences enable the clear identification of taxonomy and phylogeny [53], despite amplification bias. In addition, some studies have reported molecular analysis of microbial community structures with no replication and randomization for experimental design [30,54,55]. Nevertheless, owing to the sequencing cost or analysis techniques that need computing ability, most previous studies have described the microbial community structures from various environments, including heavy-metal-contaminated soils, using a partial length of the 16S rRNA gene sequenced by other NGS platforms. In response, this study sought to analyze the microbial community structure and estimate the relationship between microbial compositions and environmental parameters in heavy-metal-contaminated soils using a full-length bacterial 16S rRNA gene sequenced by the PacBio RS II platform with plentiful replicates for each studied site.

A number of studies have reported that environmental parameters can affect the microbial community structure and chemical processing (i.e., nutrient cycles) of soil. In the present study, we found that environmental factors influence microbial compositions (Figure 3 and Figure S3). In particular, heavy metals have significantly higher impact than other factors on microbial community structure. Contrary to the NMDS results, the CCA results showed that the microbial communities from Hwaseong are closely formed (Figure 1 and Figure S3). In addition, we identified that EC (ds m^-1^) has a higher positive correlation with heavy metals than with other environmental factors (Figure 3 and Figure S3). Although organic matter may influence the retention of heavy metals [56,57], we found no significant correlation with heavy metals (Table S1). In fact, soil EC has been known as an indirect indicator of soil health. It affects yields and suitability of crop, and plant nutrient availability, as well as key soil processes such as the emission of greenhouse gases (e.g., nitrogen oxides and methane) [58,59]. Some studies proposed that soil EC is a major factor that contributes to bacterial community and activity [60,61]. Moreover, Jordán et al. [62] contended that EC, related to metal bioavailability, shows higher correlation with heavy metal distribution. The associated network analysis conducted in the present study revealed that EC had a direct, positive relationship only with Pb and As; however, EC was also found to have an indirect, positive relationship with Cu and Cd. In addition, the aforementioned heavy metals and EC exhibited more relationships, including a negative association with microorganisms, than other environmental factors. These findings suggest that EC is a major factor associated with the activities of microbial communities (see Figure 3). Additionally, we observed that only four genera (*Acidothiobacillus*, *Leptosprillum*, *Sulfobacillus*, and Gram-positive iron-oxidizing_acidophile) exhibited a positive relationship with EC. Although the members of these genera are known to be acidophiles, our analysis did not reveal any relationship between pH and these microorganisms. Therefore, this finding possibly indicates that, compared to other environmental factors, EC has a stronger relationship with the acidophiles. Collectively, these results indicate that metal concentrations alone do not reflect toxicity or environmental pollution. Measurement and analysis of EC and heavy metal concentration can be used to effectively assess contamination risk [62] and predict indigenous microbial activity [61].

With respect to the microbial composition, Proteobacteria, Acidobacteria, and Actinobacteria phyla were identified as predominant groups (Figure 2a and Figure S1a). Notably, these phyla are commonly found in terrestrial environments [63,64,65]. However, although Acidobacteria are considered to be dominant in soil, comprising an average of 20% of total soil bacteria, the ecological roles of the phylum are still poorly understood, due to the lack of cultured representatives references in [66].

At the genus level, 16 genera were identified in this study as generalist or specialist, which were dominant. Among the genera, 10 (*Gallionella*, Gram-positive iron-oxidizing_acidophile, *Halothiobacillus*, *Leptolyngbya*, *Leptospirillum*, *Rhodoferax*, *Sulfobacillus*, *Thioalkalivibrio*, *Thiobacillus*, and YNPFFP6 classified into the family *Sulfobacillaceae*) were identified as specialist for their habitat. This suggests the possibility of the development of a microbial indicator of contamination by heavy metals in soil. Interestingly, with the exception of samples from Bonghwa, the microbial compositions of surface and subsurface samples were similar. This might be because the physiochemical characterizations of surface and subsurface soil at Daegu and Hwaseong were similar (Table S1), indicating that environmental factors affect microbial community structures. In addition, some microorganisms in BF and BB were identified as unique compared to other samples. For example, *Leptospirillum* and *Thiobacillus* were only identified as predominant in BF and BB, respectively (Figure 2b). Generally, *Leptosprillum* has been identified as an iron oxidizer and acidophile under oxic conditions [7]. It has been classified into four groups [67]; in particular, group IV is reported to be capable of hydrogen and iron oxidation [67]. Moreover, *Leptosprillum* spp. contributes significantly to AMD processing and bioleaching [7,68]. Since first being described in 1904, *Thiobacillus* spp. are known as autotrophic sulfur oxidizers, using reduced sulfur compounds as an energy source [69]. Interestingly, we found that *Halothiobacillus* was identified only in BB. Also, *Halothiobacillus* has been shown to be a minor group in AMD environments [70]. In addition, some *Halothiobacillus* species isolated from hydrothermal vents are described as halotolerant and reclassified from the genus *Thiobacillus* [71]. However, recent investigation indicated that *Halothiobacillus* might be considered an important early indicator in acid mine drainage [72]. Some members of the genus *Halothiobacillus* might be adapted to play a role in t he sulfur cycle through sulfur oxidation metabolism in a metal mine environment. For this reason, the physiological and genomic characterization of the genus *Halothiobacillus* might be worth further investigation.

On the other hand, the genus *Rhodoplanes*, described as a primarily phototrophic purple nonsulfur bacterium [73], was only found in samples from Daegu and Hwaseong with high abundance (ranging from 8% to 15% of total bacterial abundance). In addition, we found sequences related to genera that are key players in geochemical cycling for nitrogen (i.e., *Nitrospira*) or sulfur (*Sulfobacillus, Desulfosporosinus*). Unexpectedly, in addition to microbial community analysis, PICRUSt indicated relatively little functional variation between sampling sites. Only a few functional categories were observed to exhibit variation between sampling sites. The “transport” category was identified as dominant in all studied sites with a similar proportion. However, it is possible that our PICRUSt results support the relationship between microbial communities and environmental factors. Moreover, we hypothesize that the microorganism(s) are adapted to their local habitat and microbial community stability is affected during a long period of heavy-metal contamination, which acts as a selective pressure [74,75]. Based on the present analysis, we observed that EC might have a greater effect on the microbial community structure compared to heavy metals or organic matter. 

Although we successfully characterized the microbial distribution and their predicted functional traits from each sampling site, clear ecological roles and whole-metagenome analysis are lacking. Nevertheless, using full-length sequencing application, this study provides accurate information about microbial community structures and interactions in heavy-metal-contaminated soil in Korea. In addition, the findings might also enable identification of the rare biosphere [28] using full-length sequencing. For example, in DB and DF samples (Figure 2b), sequences classified in the genus *Conexibacter* were detected. To date, in the genus *Conexibacter*, only two species have been isolated as novel representatives of the deep branch of the phylum Actinobacteria from soil [76,77]. Moreover, *Conexibacter* spp. have been recently recognized in environmental metagenomics [78,79] and massive sequencing [80], including Oxford Nanopore MinION [81]. Finally, the findings of the present study provide valuable insight into the decrease in microbial activity and diversity variations caused by heavy metal pollution, as determined by full-length sequencing. 

## Figures and Tables

**Table 1:** An overview of the soil samples and estimates of 16S rRNA gene sequence diversity and phylotype coverage of the PacBio data. Diversity was estimated using operational taxonomic units (OTUs). Diversity indices and richness estimators were calculated using the Mothur package (the mother project; http://www.mothur.org).

Sample*	Grouping name	No. of analyzed reads	OTU	Chao	Shannon	Simpson	Good’s coverage	Sampling site (GPS, altitude)
BB1	BB	2738	388	1629.03	3.79	8.62	0.89	Bongwha (129°3’17.9"E, 36°51′45.6″N, 630 m)
BB2		2931	544	2092.42	4.65	24.99	0.87	
BB3		2240	355	1814.62	3.98	13.51	0.88	
BB4		2404	600	4411.03	4.14	5.74	0.78	
BB5		7769	1492	7657.55	4.66	9.77	0.84	
BF1	BF	5677	486	2820.18	2.73	3.67	0.93	
BF2		4606	508	2393.16	4.01	12.06	0.92	
BF3		4420	547	2592.36	4.07	12.18	0.91	
BF4		3969	743	2787.00	5.25	53.47	0.87	
BF5		3784	601	3295.08	4.64	24.51	0.88	
DB1	DB	4107	792	2427.76	5.03	16.91	0.87	Daegu (128°40’18.6"E, 35°46′52.5″N, 243 m)
DB2		2149	424	1513.36	4.30	8.74	0.86	
DB3		5250	1036	4241.84	5.09	13.70	0.86	
DB4		2762	551	1756.51	4.38	7.55	0.86	
DB5		8284	952	3784.81	4.99	40.10	0.92	
DF1	DF	5962	699	3354.82	4.17	13.56	0.91	
DF2		5489	712	2880.12	4.15	8.73	0.91	
DF3		4019	711	3157.89	4.39	8.37	0.87	
DF4		3880	702	2803.69	4.13	5.05	0.87	
DF5		3521	478	1580.64	4.69	36.54	0.91	
HB1	HB	4712	2425	9038.20	7.57	627.07	0.61	Hwaseong (126°55’47.6"E, 37°13′09.1″N, 105 m)
HB2		4634	2167	7723.74	7.30	355.46	0.65	
HB3		4489	2308	8079.12	7.52	557.24	0.61	
HB4		3825	2057	6954.94	7.50	521.60	0.59	
HB5		3360	1954	7019.94	7.60	726.93	0.55	
hf1	HF	3142	1673	5914.55	7.30	539.23	0.60	
hf2		1662	1026	4310.03	7.09	348.38	0.51	
hf3		3720	2097	8645.22	7.57	607.90	0.56	
hf4		1490	1032	3987.65	7.42	861.26	0.45	
hf5		5707	2801	10771.69	7.62	661.09	0.63	

* Samples were named as follows: sampling site, depth [B: subsurface (below 60–75 cm), F: surface (below 2–15 cm)] and sampling replicates.

## Supplementary Materials

The following are available online at https://www.mdpi.com/2076-2607/7/9/357/s1.
