# Supplementary material for: Identification of Microbial Profiles in Heavy-Metal-Contaminated Soil from Full-Length 16S rRNA Reads Sequenced by a PacBio System
Source: Microorganisms. 2019 Sep 16;7(9):357. doi: 10.3390/microorganisms7090357 (PMC6780547; doi:10.3390/microorganisms7090357)
Supplement: Supplementary file 1 [file microorganisms-07-00357-s001.zip › supplementary data-revised.docx]

**Supplementary Information**

**Identification of microbial profiles in heavy-metal-contaminated soil from full-length 16S rRNA reads sequenced by a PacBio system**

Moonsuk Hur^1^ and Soo-Je Park^2^*

^1^Microorganism Resources Division, National Institute of Biological Resources, 42 Hwangyeong-ro, Incheon 22689, Republic of Korea

^2^Department of Biology, Jeju National University, 102 Jejudaehak-ro, Jeju 63243, Republic of Korea

***Correspondence:**

Tel.: +82-64-754-3524; Fax: +82-64-756-3541; E-mail: sjpark@jejunu.ac.kr

**Running title:** microbial community analysis by PacBio system

**Keywords:** Heavy metal, soil, PacBio, 16S rRNA gene, next-generation sequencing, mine

**Fig. S1.** The abundance of the bacterial phyla (a) and genera (b) in the individual samples. The bacterial 16S rRNA gene sequences were assigned to each phylum using the mothur package and a reference database of 16S rRNA genes obtained from the Greengenes Database (version gg_13_8_99).

(a)

(b)


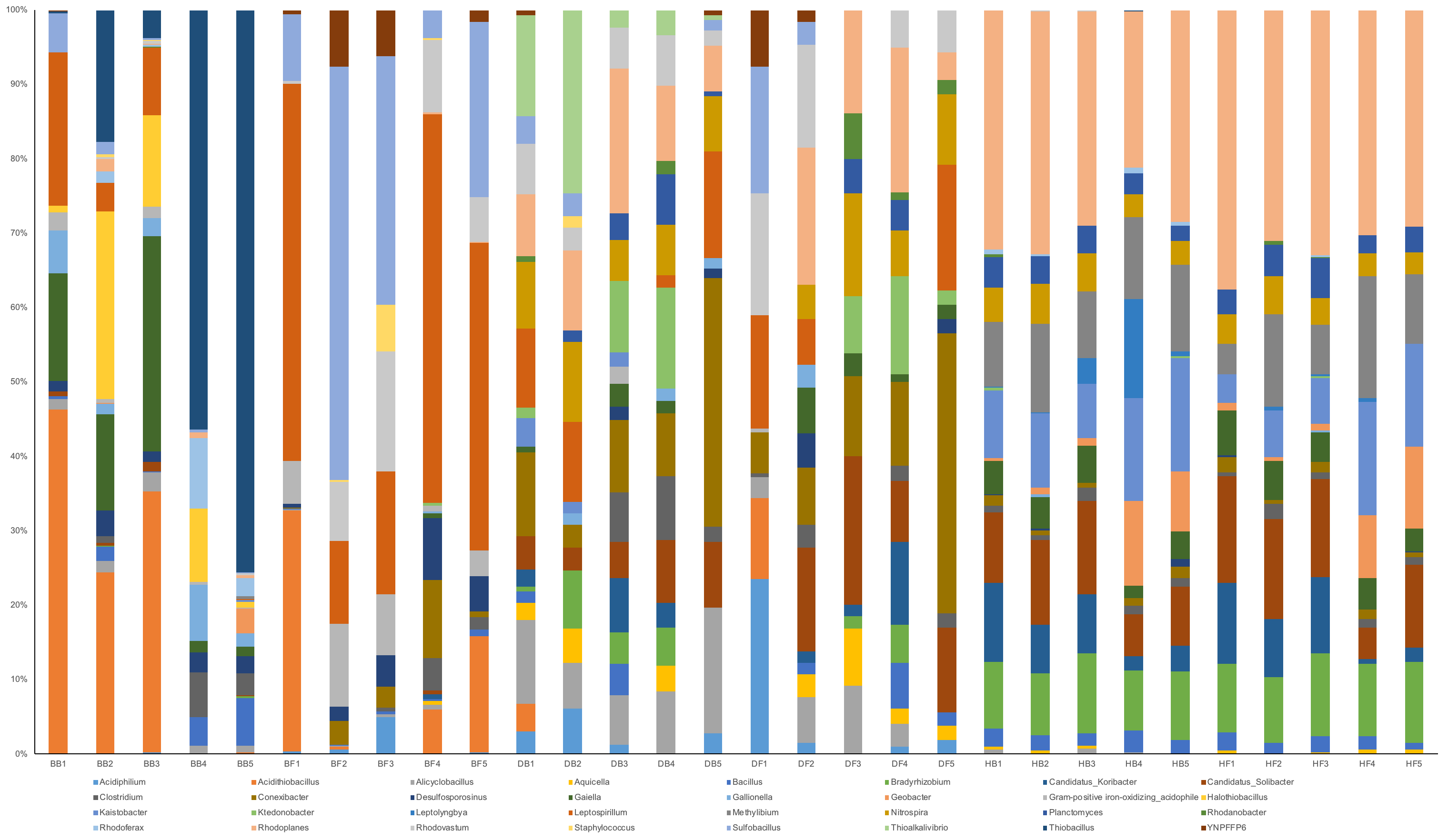


**Fig. S2.** The relationships between the microbial community profiles of the samples, represented by an unweighted pair group method with arithmetic mean (UPGMA) clustering tree. The scale bar represents the number of substitutions per site.

**Fig. S3.** Effect of environmental parameters on microbial communities and compositions by the selected genus level (above 5% of total bacterial abundances) of all individual samples, as examined using the next-generation sequencing method. A canonical correspondence analysis (CCA) ordination diagram of microbial communities associated with the following environmental variables: pH, temperature (Temp), total nitrogen (TN), total carbon (TC), carbon/nitrogen ratio (C.N), organic matters (OM), electrical conductivity (EC), heavy metals (Pb, Cd, As, Zn, and Cu), and soil compositions (Silt, Clay, and Sand).


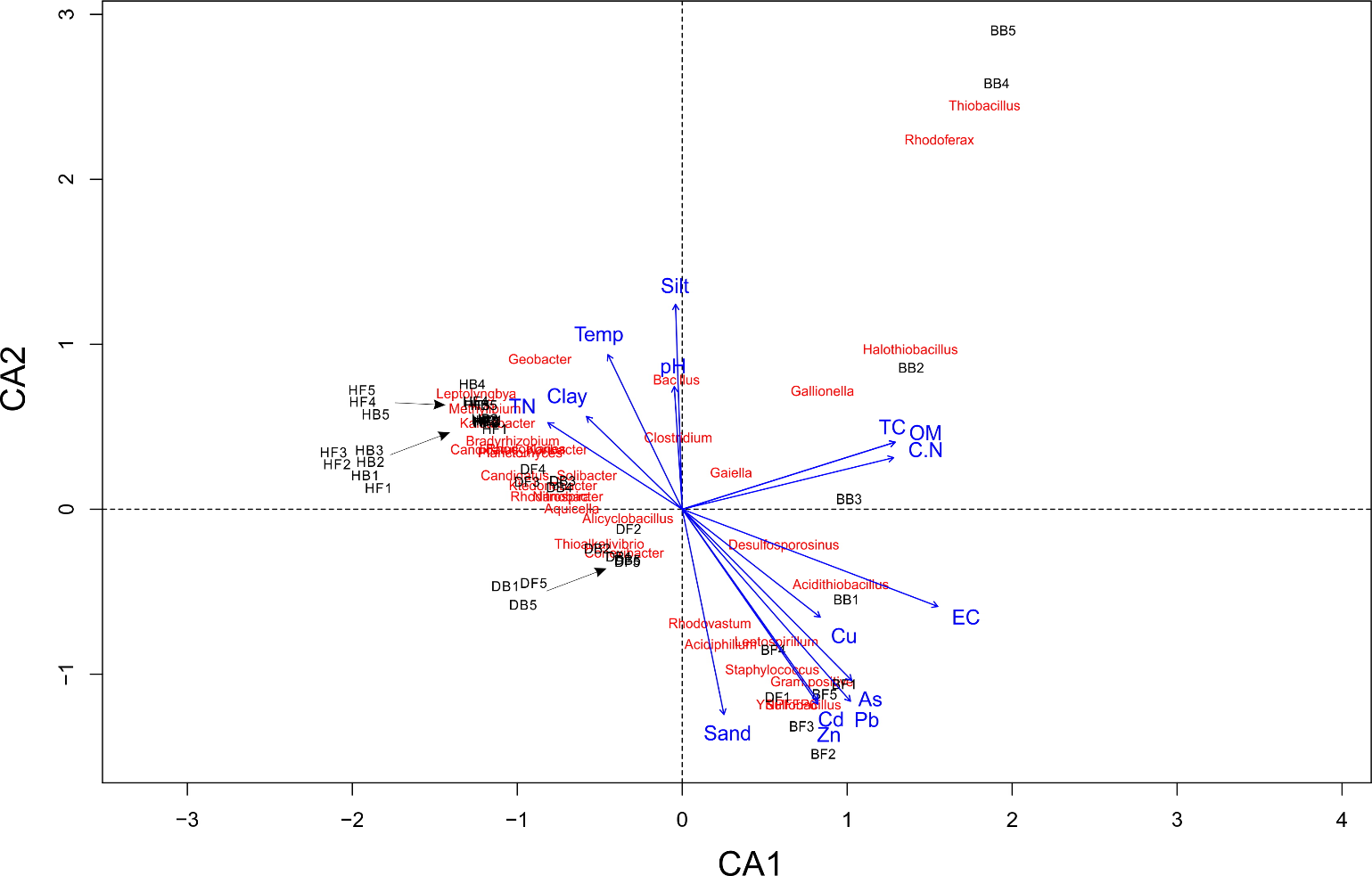


**Fig. S4.** Box plots of the operational taxonomic units (OTUs) (a), Shannon (b), Chao (c), and Simpson indices of the all samples combined by each site, H, D, and B denote Hwaseong, Daegu, and Bonghwa, respectively.

**Fig. S5.** Inferred functional profiles analyzed by PICRUSt from microbial communities of Hwaseong (named as H), Daegu (named as D), and Bonghwa (named as B). Relative abundances of KEGG categories related with Xenobiotics biodegradation and metabolism, Energy metabolism, and Membrane transport.
